# Supplementary material for: Analysis of maternal and newborn training curricula and approaches to inform future trainings for routine care, basic and comprehensive emergency obstetric and newborn care in the low- and middle-income countries: Lessons from Ethiopia and Nepal
Source: PLoS One. 2021 Oct 28;16(10):e0258624. doi: 10.1371/journal.pone.0258624 (PMC8553030; doi:10.1371/journal.pone.0258624)
Supplement: S1 Questionnaire — (DOCX) [file pone.0258624.s001.docx]

**S1 Questionnaire**

| **Data Extraction Template for Audit of Training Packages.** |
| --- |
| **Introduction** |
| Name of country (Full name): |
| Name of training manual (Full name) |
| Training course duration (write in days) |
| Which health worker cadre is eligible for the training? |
| **Training Planning and Management** |
| Are there specific instructions on ensuring appropriate training materials are available? |
| Is there a trainer’s/ facilitators guide? |
| Is there a participant’s handbook/ manual? |
| Is there a reference manual? |
| Is there a criterion for selection of participants? |
| Are there guidelines for classroom selection? |
| Are there guidelines for using clinical sites for skills-based trainings? |
| Are there guidelines for selecting appropriate trainers? |
| Were the trainers certified as master trainers? |
| Are there guidelines for improving the learning environment for trainers? For e.g.: managing group, dynamics, stress, learners’ enthusiasm? |
| **Post-Training Dimension** |
| Were the participants informed that they qualified from the course? |
| Is there a post-training action plan developed for the participants? |
| Was there a training course evaluation done? |
| Were the results of the training course evaluation ever documented and shared? |
| **Training Design-Related Questions** |
| Standardization of training activities? |
| Have the learning activities been broken down into steps for e.g.: is there a checklist, flow chart? |
| Are the assessment questions aligned to the learning objectives? |
| Are there appropriate knowledge-based activities for teaching for e.g.: readings, lecture, case study, brainstorming, facilitated discussions, group activities, games? |
| Are there appropriate skills-based activities for teaching such as skills demonstrations, simulated practice, skills practice, clinical simulation, case study, structured observation, role play, and video? |
| Are there appropriate attitude related learning activities such as behavior modelling, case study/ role play, self-reflection, journaling, client provider interaction and discussion? |
| **Activities for Assessment** |
| Are there appropriate knowledge assessment activities for teaching such as case studies, quizzes and tests, OSCE? |
| Are there appropriate skills-based assessment activities for teaching/ learning such as clinical drill coaching, direct observation, OSCE, Self-assessment, checklists? |
| Are there appropriate attitude assessment activities such as structured observations, role-play, self-reflection and assessment? |
| **Questions Related to Knowledge and Skills Assessment** |
| Was there a pre-course knowledge assessment? |
| Was there a final course assessment? (mid-course assessment) |
| Was there supervised clinical practice? |
| Was there an interim skills assessment? |
| Was there a final skills assessment? (mid-course assessment) |
| **INTERVENTION SPECIFIC QUESTIONS** |
| **Routine care during labor and childbirth** |
| **Routine care Interventions for the mother** |
| **Monitoring of labor using a partograph** |
| What is a partograph? |
| What is an alert line? |
| What is an action line? |
| Does the training recommend monitoring of blood pressure 4 hourly or more frequently in case of Pre-eclampsia? |
| Does the training recommend monitoring of temperature 2 hourly, more frequently, if febrile? |
| Does the training recommend monitoring volume of urine every time when passed and checking urine for protein and acetone? |
| How frequently should the FHR be recorded in active labor (records fetal heart rate every half hour) |
| Does the training recommend performing a vaginal examination to note the level of fetal head in relation to the ischial spines and its molding? |
| Does the training recommend assessing cervical dilatation accurately every four hours? |
| Does the training recommend assessing the color of amniotic fluid? |
| Does the training recommend monitoring the number and strength of contraction every half hour? |
| Do the training cover actions to be taken if action line is reached? |
| Does the training cover when to refer patient or start oxytocin if the labor is not progressing as expected? |
| Does the training cover recognizing abnormal fetal heart rate and taking appropriate action? |
| Does the training cover recognizing maternal condition (high pulse rate, BP, temperature, protein and/or acetone in urine, and takes appropriate action)? |
| **Infection Prevention Measures** |
| Does the training cover the importance of infection prevention and control? |
| Does the session describe infection prevention practices? |
| Does the session describe the six cleans during labor and delivery? |
| Demonstrate appropriate hand hygiene practice? |
| Demonstrate use of personal protective equipment’s and antisepsis procedures? |
| **Active Management of the Third Stage of Labor (AMTSL)** |
| Does the training describe the anatomy of the uterus? |
| Does the training explain the natural physiological process of placental delivery? |
| What is the definition of AMTSL? |
| Are there opportunities to describe and demonstrate AMTSL? |
| What is the dose of the uterotonic for AMTSL? |
| Does the training cover examination of the placenta and membranes for completeness? |
| Does the training explain available research evidence to support AMTSL? |
| Does the training explain why AMTSL can save lives? |
| Does the training describe the warning signs for complications that may arise during the third stage of labor? |
| **Newborn Care – Routine Care** |
| **Thermal protection** |
| Does the training cover thermal protection such as ensuring that room temperature is warm, eliminating drafts from open windows / doors / fans etc. for the arrival of the baby? |
| Does the training cover thoroughly drying the baby immediately after birth? Wipes the face and dries the baby thoroughly immediately after birth and discards the wet cloth? |
| Does the training cover explaining to the mother about the benefits of skin-to-skin contact for thermal protection? |
| Does the training include keeping babies in skin-to-skin contact (with body and head covered) with their mothers for at least one hour after birth? |
| If the room is cool (<25 ºC), uses a blanket to cover the baby over the mother. |
| If the newborn is small, encourages the mother to keep the newborn in skin-to-skin contact for as long as possible, day and night. |
| Advise mother and family to continue practicing KMC at home |
| **Immediate and Exclusive Breast Feeding** |
| Does the training cover counselling the mother and her family on how breastfeeding helps mothers and babies? |
| Does the training explain the benefits of early and exclusive breastfeeding? |
| Does the training cover supporting and guiding a mother to initiate and maintain successful first breastfeeding? |
| Does the training assess whether the mother is breastfeeding well? |
| Does the training cover how to support a mother attach a baby to her breast? |
| Does the training explain to the mother about the optimal pattern/timing of breastfeeding? |
| Does the training cover counseling HIV positive mother on infant feeding? |
| Does the training cover recognizing and caring for common breastfeeding problems? |
| **Neonatal infection prevention including hygienic cord care** |
| Does the training cover ensuring strict hand washing before and after handling babies |
| Does the training cover importance of rooming in with the mother? |
| Does the training cover co-bedding of mother and infant (use of a heated cot as required & minimum use of incubators)? |
| Does the training encourage breast feeding (less need for Parenteral feeding)? |
| Does the training mention overcrowding in the labor room/ wards/ NICU as sources of infection? |
| Does the training cover importance of using clean water? |
| Does the training cover importance of hospital staff washing hands? |
| Does the training cover the infection prevention approaches during labor and childbirth? |
| Does the training mention that late cord clamping (performed after one to three minutes after birth) is recommended for all births while initiating simultaneous ENC? |
| Does the training mention appropriate cord care? |
| **Preparedness for Neonatal Resuscitation** |
| Does the training ensure that health workers check that equipment and supplies are available and functioning? |
| Does the training mention having an extra pair of hands to support with neonatal resuscitation if required? |
| Does the training cover provision of immediate ENC at birth? |
| Does the training cover thorough drying at birth and rubbing the back as stimulation to help the baby breathe? |
| Does the training cover how to determine which baby requires resuscitation? |
| Does the training provide opportunity to practice steps of resuscitation on a model? |
| Does the training cover positioning/ open the airway by extending the neck? |
| Does the training cover not suctioning all babies and only for those who have secretions blocking their mouth and nose or have meconium in amniotic fluid? |
| Does the training cover how to support the mother and the family? |
| **Basic Emergency Obstetric and Newborn Care** |
| **Maternal care interventions for BEmONC** |
| **Parenteral magnesium sulphate for (pre-) eclampsia** |
| Outline diagnostic criteria for pre-eclampsia |
| Classify pre-eclampsia according to severity |
| Outline risk factors for pre-eclampsia |
| Outline maternal and fetal complications of pre-eclampsia. |
| Does the health worker counsel the woman and the family about the need for treatment, how it works and explain the need for referral to the higher center if complication management is not available? |
| Has the health worker been taught to make a rapid evaluation of the general condition of the woman including vital signs (pulse, blood pressure, respiration) while simultaneously finding out the history of her present and past illnesses either from her or from her relatives? |
| If she is not breathing or her breathing is shallow: assists ventilation using bag and mask or gives oxygen at 4–6 L per minute via nasal catheter? |
| If she is unconscious, checks airway and temperature; position her on her left side; check for neck rigidity. |
| If she is convulsing: - positions her on her left side to reduce the risk of aspiration of secretions, vomit and blood; - protects her from fall and injuries; puts on air way; provides constant supervision; |
| If eclampsia is diagnosed initiates magnesium sulphate as per clinical guidelines; if the cause of convulsions has not been determined, manages as eclampsia and continues to investigate other causes |
| Sets up IV drip using standard guidelines. Loading dose: gives 4gm magnesium sulphate as 20% solution IV over 5 minutes; and 10g of 50% magnesium sulphate solution, 5 g in each buttock as deep IM injection, with 1mL lignocaine 2% |
| Gives magnesium sulphate 5g (50% solution) + 1mL lignocaine 2% IM every 4 hours into alternate buttocks |
| What are the prerequisites for giving maintenance dose of MgSO4 (the respiratory rate is at least 16 per minute, patellar reflexes are present, urinary output is at least 30 mL per hour over 4 hours)? |
| If facilities/skills for intubation and caesarean section are not available ensures that the woman is referred to a higher center after giving the loading dose of magnesium sulphate and having stabilized her blood pressure and general condition. |
| Performs obstetric examination to confirm fetal position, presentation, heart rate and whether the woman is in labor |
| If labor is advanced, manages as per the standard labor management protocol, avoids giving bolus dose of Ergometrine when maternal blood pressure is high and if she has eclampsia |
| **Assisted Vaginal Delivery** |
| Gets help of another colleague to receive the baby and provide necessary support including operating the vacuum pump if it is hand operated |
| Prepares necessary equipment |
| Explains to the woman and her support person what is going to be done, listens attentively and respond to her questions |
| Provides continual emotional support and reassurance, explaining the process as it progresses? |
| Checks the gestation period to ensure that the pregnancy is not preterm? |
| Examines the fetal heart to ensure that the baby is alive and conducts abdominal palpation to ensure that the head is less than 2/5th palpable |
| Performs vaginal examination to ensure that the fetus is presenting by the vertex, cervix is fully dilated, head is at or below the ischial spines with little or no molding and the membranes are ruptured? |
| Ensures the bladder is empty |
| Checks the equipment to ensure that the vacuum is satisfactorily created and maintained |
| Prepares for delivery using aseptic method |
| Does a vaginal examination to reconfirm the position of sutures and fontanelles and applies cup accurately as per the standard guideline, ensures that vacuum is created, vaginal tissue is not pulled into the cup and applies the correct negative pressure. |
| Delivers baby using gentle traction and applies episiotomy, as necessary? |
| Delivers placenta using AMTSL? |
| Examines cervix and vagina for any tear and repairs tear |
| Ensures baby gets immediate ENC |
| **Parenteral Antibiotics for Maternal Infection** |
| What are infections during pregnancy? |
| What are risk factors for infection during pregnancy? |
| What are the routes of infection? |
| How do you define maternal sepsis? |
| What are the signs and symptoms of maternal sepsis? |
| What is the therapeutic protocol for administration of parenteral antibiotics for maternal sepsis? Give Ampicillin 2g I/V every 6 hours PLUS Gentamicin 80mg im or by slow i/v injection every 8hrs PLUS Metronidazole 500mg every 8hrs |
| What is the regimen for prophylactic antibiotics: Caesarean Section Either Augmentin 1.2 G iv or Cefuroxime 1.5 g iv and Metronidazole 500 mg iv after clamping the cord |
| Do the health workers counsel the mother or the family on infections during pregnancy? |
| Is there a discussion on good practices for IM and IV administration of antibiotics? |
| **Parenteral Oxytocic Drugs for Hemorrhage** |
| Palpates the uterus to make sure no other baby is present |
| If no other baby is present, administers uterotonic drug (oxytocin 10 IU IM is the uterotonic of choice) within one minute of delivery (if a woman has an IV infusion, an option is giving oxytocin 5 IU IV bolus slowly). |
| The health worker knows that much lower dose is to be used for induction or augmentation of labor. Initial dose: 0.5 to 1 milli-units IV infusion per hour. At 30 to 60-minute intervals the dose should be gradually increased in increments of 1 to 2 milliunits until the desired contraction pattern has been established. |
| The health worker knows that high dose of oxytocin can lead to fetal distress, and or rupture of the uterus |
| **Manual Removal of Placenta for Retained Placenta** |
| Does the training cover when do you have to manually remove the placenta? |
| How do you provide emotional support and counselling to mother/family prior to MRP? |
| Does the training cover giving an effective anesthesia/analgesia prior to MRP? |
| Does the training cover giving a single dose of prophylactic antibiotic? |
| Does the training cover aseptic precautions to be taken prior to MRP? |
| Does the training cover the technique to manage a retained placenta? |
| Is the placenta checked for completeness so that any further exploration of the uterus may be carried out without delay? |
| Is an uterotonic drug given after completion of the placenta? |
| What are the complications following the procedure? |
| **Removal of Retained Products of Conception** |
| What do you mean by retained products of conception? |
| What are the signs and symptoms that a patient presents with? |
| Does the health worker assess patient’s condition for shock or other complications? |
| Does the training cover how to counsel the woman and her support person? |
| Does the training mention that the health worker should explain each step of the procedure as being carried out? |
| Does the training cover techniques to perform bimanual examination? |
| Does the training cover providing anesthesia (paracervical block)? |
| Does the training provide details on the MVA procedure? |
| Does the training provide information on how to check for signs of completion (red or pink foam, no more tissue in cannula, a “gritty” sensation and uterus contracts around the cannula) |
| Does the training cover details about post procedure tasks such as maintaining asepsis, hand-hygiene, and safe disposal of sharps |
| Are providers taught to ensure that there is no bleeding post procedures and that the patient doesn’t have any cramps? |
| Does the training advise health workers to provide family planning counselling and commodity |
| **Antibiotics for Preterm or Prolonged PROM to Prevent Infection** |
| What is Preterm premature rupture of membranes? |
| Why is PROM IMPORTANT? |
| What are the components of history taking for PROM? |
| How do you conduct a physical examination for PROM? |
| How do you diagnose PROM? |
| What are the recommendations for giving antibiotics to patients with PROM based on gestational age? |
| What are the complications of PROM? |
| **Antenatal Corticosteroids in Preterm Labor** |
| What are the benefits of antenatal corticosteroids? |
| How to counsel the woman about the advantages of corticosteroid when preterm birth is imminent between 24 to 34 weeks? |
| Does the protocol encourage women, if possible, to deliver in appropriate facilities that provide adequate childbirth care, including preterm newborn care? |
| At what gestation, should ACS be used? |
| How long after administration is a course of corticosteroids most effective? |
| How to rule out any maternal infection before giving corticosteroids, makes sure there is no chorioamnionitis in case of premature rupture of membranes |
| What is the recommended treatment protocol? Intramuscular (IM) dexamethasone or IM betamethasone (total 24 mg in divided doses) is recommended as the ACS when preterm birth is imminent. |
| What is the protocol for giving tocolytics to make ensure that the labor does not progress till corticosteroids get time to act? |
| Does the treatment protocol ensure that a single repeat course of antenatal corticosteroid is recommended if preterm birth does not occur within seven days after the initial dose, and a subsequent clinical assessment demonstrates that there is a high risk of preterm birth in the next 7 days? |
| Does the protocol allow administration of antibiotics if there is PROM (without infection) but not when the membranes are intact? |
| **Newborn Care Interventions for BEmOC** |
| **Resuscitation with Bag and Mask of Non-Breathing Baby** |
| What is the protocol for providing resuscitation? |
| How do you position/ open the airway by extending the neck? |
| How do you clear airway if meconium is present |
| What to do if meconium is present and the baby is pink, crying and has a good tone? |
| How do you provide positive pressure ventilation with bag and mask? |
| What are the indications for bag and mask ventilation? |
| How do you know if the baby is improving and that you can stop positive pressure ventilation? |
| What do you do if baby’s chest is not moving during bag and mask ventilation? |
| What are the indications for suction? |
| What should you do after a baby has been successfully resuscitated? |
| How do you identify birth asphyxia? |
| **Kangaroo Mother Care for Premature/Very Small Babies** |
| How to counsel the mother and the family about the benefits of KMC for a low birth weight baby? |
| What is the criteria for initiating KMC? (weight less than 2500 grams, stable cardio-respiratory condition, ability to suck and swallow) |
| What is the position of the baby for KMC? *(Place the baby in skin-to-skin contact between the mother’s breasts with the baby’s feet below her breasts and the baby's hands above with arms flexed like a "frog position", positions the baby's head correctly and ensures that it is well supported)* |
| Ensures baby's back is covered with extra clothing to keep her warm |
| Ensures baby's wet diaper is frequently changed, especially if a cloth diaper is used to prevent soiling of the mother's skin |
| Baby is nicely maintained in skin contact with the mother by an extra sheet that is wrapped around the baby and the mother |
| Does the health worker have opportunities to practice with the mother and the care giver until they are confident |
| Does the health worker encourage the father and other family members to support the mother and provide alternative KMC support if mother needs a break |
| Do the training materials promote breast feeding for the premature, low birth weight infant and providing necessary support to establish breast feeding or to feed using expressed breast milk? |
| **Alternate Feeding if Baby is not Able to Breastfeed** |
| How to express breast milk for cup feeding/ alternate feeding? |
| Counsels mother on how to express breast milk |
| When to cup feed a baby? *Cup feeding should be used for babies who are able to swallow but not able to feed adequately from the breast. When using an alternative method to feed breast milk* |
| How to cup feed a baby? |
| Assess the baby’s ability to take cup or spoon feedings. |
| **Injectable Antibiotics for Neonatal Sepsis** |
| Knowledge and skills to determine if urgent referral is needed using “assess and classify” the sick infant chart. *(Referral for the following conditions: Possible Serious Bacterial Infection, Severe Jaundice, Severe Dehydration, Severe Persistent Diarrhea, Severe Dysentery, Not Able to Feed - Possible Serious Bacterial Infection or Severe Malnutrition)* |
| Are health workers able to identify treatments for patients who need urgent referral? |
| What is the recommended protocol for providing urgent pre-referral treatments? [Give first dose of intramuscular injection of Ampicillin and Gentamicin, treat to prevent low blood sugar, Warm the young infant by Skin to Skin contact if temperature less than 36.5-degree C (or feels cold to touch) while arranging referral, Advise the mother how to keep the infant warm on the way to the hospital, advise mother to give frequent sips of ORS and continue breast feeding on the way, advise mother to continue breastfeeding] |
| Explain the need for referral of the mother and writing the referral note |
| Identify Treatments for Young Infants Who Do Not Need Urgent Referral |
| What are the four steps to refer a young infant to the hospital? |
| Explain to the mother the need for referral and get her agreement to take the young infant. In addition, explain that young infants are particularly vulnerable. When they are seriously ill, they need hospital care and need to receive it promptly. If you suspect that she does not want to take the infant, find out why. |
| Calm the mother's fears and help her resolve any problems. |
| Write a referral note for the mother to take with her to the hospital. Tell her to give it to the doctor there |
| Give the mother any supplies and instructions needed to care for her infant on the way to the hospital? |
| Are there enough sections on determining appropriate oral drugs and dosages for a sick young infant? |
| Are there enough sections on providing oral antibiotics and teaching the mother how and when to give them at home such as for treating local infections (such as umbilical or skin infections, ear drainage and thrush), and teaching the mother how and when to give the treatments at home? |
| Checking a mother's understanding of neonatal sepsis? (Danger signs) |
| Giving drugs administered in the clinic only (intramuscular injections of ampicillin and gentamicin)? |
| Warming the young infant who has temperature less than 36.5 degrees or feels cold to touch? |
| Preventing low blood sugar? |
| Treating different classifications of dehydration, and teaching the mother about extra fluid to give at home? |
| Teaching the mother to treat breast and nipple problems and correct positioning and attachment? |
| **Management of the HIV Exposed Infant** |
| What are the components of PMTCT programs? *[preventing new HIV infections among women of childbearing age; preventing unintended pregnancies among women living with HIV; preventing HIV transmission from a woman living with HIV to her baby; providing appropriate treatment, care and support to mothers living with HIV and their children and families]* |
| What are the additional things to keep in mind for the immediate care and management of the HIV born infant (*Where mothers are AFB sputum positive (have active TB and are on treatment), BCG vaccine should NOT be given to infants until they have completed 6 months of INH prophylaxis. • Infants of mothers who are Hepatitis B surface Antigen positive (HBsAg) must receive Hepatitis B immunoglobulin and Hepatitis B vaccination within 12 hours of birth.)* |
| When should infants born to HIV positive mothers receive a course of antiretroviral treatment? |
| What are the treatment recommendations for breastfeeding for infants with HIV positive mothers? *Breastfeeding - the infant should receive once-daily Nevirapine from birth for six weeks. Replacement feeding - the infant should receive once-daily Nevirapine (or twice-daily zidovudine) from birth for four to six weeks.* |
| When should infants born to HIV positive mothers be tested*? At four to six weeks old, all infants who are born to HIV-positive mothers should be given an early infant diagnosis. Another HIV test should be done at 18 months and/or when breastfeeding ends to provide the final infant diagnosis.* |
| What are the infant feeding options for HIV exposed infants- for HIV negative women and women who do not know their status? |
| What are the infant feeding options for HIV exposed infants- Feeding recommendations for infants of HIV-positive women |
| What is exclusive replacement feeding? |
| How do you monitor and counsel on exclusive feeding options and dangers of mixed feeding? |
| **Comprehensive Emergency Obstetric Care** |
| **Maternal CEmOC Interventions** |
| **Caesarean section** |
| What is the anatomy and physiology of gravid uterus and fetal wellbeing? |
| Are there skills- based sessions on conducing abdominal examination for presentation and position of fetus, clinical estimation of liquor volume and fetal heart sound; |
| What are the stages and phases of normal labor? |
| How do you conduct a vaginal examination for identifying effacement and dilation of cervix, station of the presenting part, presence and absence of membrane, adequacy of pelvis, cephalo-pelvic disproportion, caput and molding? |
| What are the elements of a partograph and their interpretation? |
| How do you plot a partograph based on examination findings and interpreting the same to take a decision on whether or not and when to perform LSCS? |
| What are the common complications during labor such as prolonged/obstructed labor, fetal distress etc.? |
| How do you recognize the signs and symptoms of prolonged/obstructed labor fetal distress etc. and its management? |
| What are the indications of LSCS? |
| How do you conduct a clinical assessment of indications for elective and emergency LSCS, including recognizing signs and symptoms of prolonged labor/ obstructed labor, fetal distress etc.? |
| What are the steps of Caesarean section operations? |
| What are the steps/precautions to be taken while incising the uterus and performing LSCS? |
| What are the elements of routine post-operative care following LSCS, potential complications during and following surgery such as PPH, Visceral injury, Internal hemorrhage, Inversion of uterus, Others? |
| How do you recognize signs and symptoms of complications following LSCS and their management including modified B-lynch suturing and obstetric hysterectomy? |
| **Newborn CEmOC intervention** |
| **Fluid Management in a Newborn** |
| Describe how maintenance fluids are calculated according to the weight and the age of the baby? |
| What are the indications for IV fluids? |
| How to monitor a neonate who is receiving IV fluids? |
| Describe when and how to introduce IV fluids if newborn is stable and feeds are not contraindicated? |
| Describe when and how to introduce IV fluids if there is a contraindication to oral fluids/ feeds? |
| **Bubble Continuous Positive Airway Pressure (b-CPAP)** |
| What is bubble C-PAP ? |
| What happens when a child has respiratory problems? |
| How does b-CPAP help? |
| When does a newborn require b-CPAP? |
| What are specific indications for b-CPAP? |
| How to set up a newborn on b-CPAP? |
| How to monitor newborn on b-CPAP? |
| How to wean a newborn off b-CPAP? |
| What are complications of b-CPAP? |
| **Safe Oxygen therapy** |
| What are the requirements for safe oxygen use in newborns? |
| What are the systems for delivering different oxygen concentrations? |
| How do you monitor safe oxygen therapy? |
