# Supplementary material for: Analysis of maternal and newborn training curricula and approaches to inform future trainings for routine care, basic and comprehensive emergency obstetric and newborn care in the low- and middle-income countries: Lessons from Ethiopia and Nepal
Source: PLoS One. 2021 Oct 28;16(10):e0258624. doi: 10.1371/journal.pone.0258624 (PMC8553030; doi:10.1371/journal.pone.0258624)
Supplement: S1 File — (DOCX) [file pone.0258624.s002.docx]

**S2 Form**

**Date: __________________**

**Name of the country: ____________________________**

**Name of the key informant: ­______________________________**

**Role within the training: Trainer/ Participant/ facility manager/ Government health official or equivalent**

**Name of training manual: __________________________**

1. What did you think of the training planning and management? (Probe: venue, seating arrangements, IT facilities, projector, meals, off-site or on site, duration, trainer to participant ratio and others)
2. What did you think of the training materials? (Probe: whether materials are user friendly, understandable, adequate time allotted for sessions, skills training, adequate copies available)
3. What did you think about the selection criteria for trainers/ participants?
4. In your opinion, how could the training have been made more effective?
5. In your opinion, does the training help participants acquire the relevant skills or reach competency in skills-based elements? Please elaborate?
6. In your opinion, does the training help participants acquire the relevant knowledge? Please elaborate?
7. In your opinion after the training, are the participants able to apply their new knowledge and skill in their day to day work?
