# Supplementary material for: Analysis of maternal and newborn training curricula and approaches to inform future trainings for routine care, basic and comprehensive emergency obstetric and newborn care in the low- and middle-income countries: Lessons from Ethiopia and Nepal
Source: PLoS One. 2021 Oct 28;16(10):e0258624. doi: 10.1371/journal.pone.0258624 (PMC8553030; doi:10.1371/journal.pone.0258624)
Supplement: S2 File — (DOCX) [file pone.0258624.s003.docx]

**S3 Form**

**KEY INFORMANT CONSENT FORM FOR INTERVIEWS**

I _________________________________________agree to be interviewed for the purposes of this research.

**I understand that:**

- I am agreeing to be interviewed regarding the quality of newborn care trainings.
- All the findings from this research are confidential and will not be linked to any individual health workers at this health facility.
- This decision has been taken voluntarily and will not have any implications for me or my health facility.
- I have been provided with the necessary information about this research and have also had an opportunity to clarify all my questions.

My questions have been answered by ­­­­­­­­­­­­­­­­­­­­­___________________________________

Signature of the key informant ­­­­­­­­­­­­­_______________________

Date: _______________________________
