# Supplementary material for: Analysis of maternal and newborn training curricula and approaches to inform future trainings for routine care, basic and comprehensive emergency obstetric and newborn care in the low- and middle-income countries: Lessons from Ethiopia and Nepal
Source: PLoS One. 2021 Oct 28;16(10):e0258624. doi: 10.1371/journal.pone.0258624 (PMC8553030; doi:10.1371/journal.pone.0258624)
Supplement: S3 File — (DOCX) [file pone.0258624.s004.docx]

## S4 Form

**INFORMATION SHEET FOR KEY INFORMANT INTERVIEWS**

**Dear Madam/Sir,**

We are conducting a study to explore the quality of trainings for essential care at the time of birth in XX country. This study has been approved by the Save the Children Ethics review Committee (ERC) and clearance was also obtained from the MOH.

**Why is this important?**

As you know, newborn health is an important priority for the government of XX. Hence, we are conducting this research to understand more about the quality of newborn health trainings offered to health workers in XX country.

**Who is carrying out the study?**

This study is funded by USAID through the MCSP project. Save the Children is facilitating the study in-country.

**What is involved?**

As a key informant, we are very keen to talk to you and hear your insights on the content, duration, methodology and your overall impression of the newborn care training.

**Is this research confidential?**

Yes. Any information obtained from this research is confidential and will only be seen by the members of the research team. All information will be stored securely. This means that any findings from the interviews will not be linked to any individual health worker.

**What are the benefits of taking part in this research?**

There are no direct benefits to you for participating in this research. However, we will use the information obtained from the interviews to improve the newborn health trainings offered in XX country.

**What are the risks in taking part?**

There are no risks as a result of taking part in this research. We will anonymize all our findings and will not be able to link your opinions to individual health workers or facilities.

**Do I have to take part in this research?**

No. If you decide not to participate in the study, it will not have any effect on you or your health facility.

**How will the research findings be used?**

The findings of the research will be used to develop a report which will highlight the existing quality of newborn health trainings in XX country. If you would like, we are happy to provide you with a summary of the findings of our research at the end of the study.

**Thank you for taking the time to read this information. We really appreciate your participation in this research.**

The interviews will only proceed once you have asked any other questions that you may have and have signed the relevant consent forms. You can keep this information sheet with you. If you have any questions or opinions about this study, please contact: ……………………………………………………….
