# Supplementary material for: Analysis of maternal and newborn training curricula and approaches to inform future trainings for routine care, basic and comprehensive emergency obstetric and newborn care in the low- and middle-income countries: Lessons from Ethiopia and Nepal
Source: PLoS One. 2021 Oct 28;16(10):e0258624. doi: 10.1371/journal.pone.0258624 (PMC8553030; doi:10.1371/journal.pone.0258624)
Supplement: Attachment — Submitted filename: Response to Reviewers 16092021[49].docx [file pone.0258624.s007.docx]

**Response to reviewers**

Reviewer #1: The authors have brought considerable changes to the manuscript and incorporated previous comments and suggestions. The below suggestions will help to improve the manuscript further.

**Response**: Thank you to the reviewers for your thoughtful and constructive comments which have helped to strengthen our manuscript further. Please find our responses below.

**Reviewer 1, Comment 1**: There are some grammatical and typographical errors in the manuscript, and the authors need to correct them.

**Reviewer 1, Response 1:** We have reviewed and revised the manuscript thoroughly for grammatical and typological errors.

Abstract:

**Reviewer 1, Comment 2:** The aim of the study seems complex. Therefore, the authors need to make the aim of the study simple, concise, and focused. Meanwhile, the integration of training packages and services stated in the first paragraph does not match the study's aim.

**Reviewer 1, Response 2:**

We have now revised the abstract to make it simpler as suggested.

**Reviewer 1, Comment 3:** Keywords: It would be better to match the selected keywords with MeSH.

**Reviewer 1, Response 3:We have now matched keywords with MeSH headings as suggested.**

Developing Countries, Health Services, Maternal Health, Newborn Health, Training, Inservice, On-the-Job, Ethiopia, Nepal.

**Introduction:**

**Reviewer 1, Comment 4:** The introduction section is unnecessarily lengthy. It would be better to keep this section brief, clear, and aligned with the objective of the study. Several paragraphs are slipping away from the study's central concern, e.g., page 5, second paragraph. In addition, the aim of the study stated in the introduction should be aligned with the abstract.

**Reviewer 1, Response 4:** We have now revised the introduction section, tightened the text and deleted repetitions. We have also aligned the aims of the study in the introduction section with the abstract as advised.

**Methods:**

**Reviewer 1, Comment 5:** The authors need to include relevant references to the selected design/methods of the study.

**Reviewer 1, Response 5:** Thanks. We have now added two references for the methods/ design section.

Results:

**Reviewer 1, Comment 6:** Figures 2 and 3 captions should be adjusted to the subheadings (page 16-18).

**Reviewer 1, Response 6:** We have adjusted the captions accordingly as suggested.

**Reviewer 1, Comment 7:** Adding some quotes from the study participants will enrich the qualitative results.

**Reviewer 1, Response 7:** Thank you for your comment. We did not put quotations initially given the word count but have now added two quotations on page 24 lines 439 onwards.

**Discussion:**

**Reviewer 1, Comment 8:** It will be better to compare the findings with other similar studies in LMIC and explain the differences.

**Reviewer 1, Response 8:** To the best of our knowledge, there is limited research evidence from peer-reviewed and grey literature systematically assessing training content and implementation experiences of in-services training programs in Nepal and Ethiopia. We have added the following to line 551 in the discussion section. *This study adds to the limited but growing evidence-base on the content of various in-service training materials and their implementation experience in both countries.*

**Reviewer #3**: I commend the authors for addressing a topic that has large programmatic relevance. Maternal and newborn health is an integral part of sustainable development targets. A huge amount of government and aid money is spent on MNH training.

Generally speaking, the manuscript is written well and followed a sound technical approach. However, there are a few areas that need explanation and/or improvement.

**Reviewer 3, Comment 1:** The authors stated that their evaluation targeted levels 1 (reaction) and 2 (learning) of the Kirkpatrick Framework but I have not seen the learning component clearly assessed in the paper. The authors should review the introduction section where they describe the Kirkpatrick evaluation framework. Levels 2 and 3 are exchanged.

**Reviewer 3, Response 1:** Kindly note that the reference to the Kirkpatrick framework was removed during the first revision of the manuscript. Hence, we can confirm that this version of the manuscript has no mention of and does not refer to the Kirkpatrick framework.

**Reviewer 3, Comment 2:** In the methods section, the authors stated that they excluded training packages that are not approved by the MOH in the focus countries. What proportion of MNH trainings followed approved training materials? How might this have affected the selection and results?

**Reviewer 3, Response 2:** Our selection criteria for inclusion was limited to collecting and analyzing existing national-level training materials which were endorsed and approved by the MoH in both countries. We did not capture information on the whole spectrum of training materials that may exist in countries and limited ourselves to those that were officially endorsed or approved by the MOH for implementation. Therefore, we are not in a position to provide any insights on other “non-approved” training materials. In addition, we validated our findings with program focal persons and key stakeholders in both countries during national consultative workshops (attended by >20 participants) and are confident that we have included all the relevant training materials.

**Reviewer 3, Comment 3:** The data extraction tool has binary responses (Yes or No). I wonder if the data extractors had difficulty in giving a Yes/No answer and what influence that might have had on the findings.

**Reviewer 3, Response 3:**

The lead researchers in both countries were experienced senior technical experts and did not have any difficulty in assigning yes/ no responses. Wherever technical content was found to be lacking compared to our data extraction tool, researchers gave that a no response. In addition, the key informant interviews and the validation workshops were used to clarify any technical nuances or specific clinical details.

**Reviewer 3, Comment 4:** The manuscript identified missing technical contents in the reviewed materials. On the other hand, the authors rightly acknowledge the concerns about integration and said that training should respond to specific performance gaps instead of universal coverage of topics. The latter suggests we should not expect every training material to cover every related topic. It is not clear how the authors determined essential from nice to know content. Lack of clarity on this can potentially affect the validity of conclusions.

**Reviewer 3, Response 4:** Thanks for this very important comment. The recommendations that emerged from this study respond to the gaps identified from the desk review, key informant interviews and consultations with national experts. On the issue of the technical scope of the training materials, our recommendations are to close down identified gaps based on the minimum technical standards or signal functions that have been identified for routine care, basic and comprehensive obstetric and neonatal care recommended by the World Health organization and other technical agencies. These signal functions are the most effective medical interventions that are essential for managing maternal and newborn health complications and will require trained health workers working in appropriately resourced facilities to provide high-quality care to the population.

**Reviewer 3, Comment 5:** There are needs for editorial improvements. Full stop at the end of sentences is missing in many places.

**Reviewer 3, Response 5:** Thank you. We have reviewed and revised the manuscript thoroughly for grammatical and typological errors including punctuations.
